# Antimicrobial resistance pattern and uropathogens distribution in children visiting a referral hospital in Mogadishu

**DOI:** 10.2144/fsoa-2023-0298

**Published:** 2024-05-15

**Authors:** Abdikarin Ahmed Mohamed, Yavuz Bastug, Cem Senol, Mohamed Muktar Kassim, Abdisalam Abdullahi Yusuf, Abdikarim Hussein Mohamed

**Affiliations:** 1 Department of Pediatrics & Child Health, Mogadishu Somalia Turkish Training & Research Hospital, Mogadishu, Somalia; 2 University of Somalia, Mogadishu, Somalia

**Keywords:** antimicrobial resistance, multidrug-resistant patterns, pediatric urinary tract infection

## Abstract

**Aim:** Studies concerning epidemiology and drug susceptibility patterns of pediatric urinary tract infection in developing countries are scarce. This study aimed to evaluate the antimicrobial resistance pattern and uropathogens distribution in children. **Method:** Four-year retrospective study included 840 participants in all pediatric age groups whose urine had been cultured. **Results:** The prevalence of culture-proven pediatric UTIs was 17.6% (148/840). *Escherichia coli* was the most common pathogen isolated from the cultures, accounting for (48%, 71/148), followed by *Klebsiella pneumoniae* (16.2%, 24/148). About 27% of the pathogens had a multidrug-resistant (MDR) pattern. A resistance rate against nitrofurantoin at 24.6%, fosfomycin at 15.2% and trimethoprim-sulfamethoxazole (SMX-TMP) at 79.7% was noted. **Conclusion:** *E. coli* and *Klebsiella pneumoniae* were the most common pathogens isolated.

## Abstract (plain language summary)

UTI is a common and important clinical issue that warrants attention among children and parents. Infants and young children may come with nonspecific signs and symptoms, including fever and fussiness and occasionally parental reporting of irritability and foul-smelling urine.

İt accounts for nearly 1% of physician office visits and 5–14% of emergency visits in children annually.

Gram-negative organisms are most commonly isolated from urine samples of children, with *Escherichia coli* accounting for almost half of the isolates.

High rate of multidrug-resistant organisms were identified, it is therefore suggested that there has to be a high index of suspicion and appropriate antimicrobial selection to reduce the risk of MDR and improve the effectiveness of antibiotics.

Urinary tract infection (UTI) is the third most common pediatric infection after respiratory and GI tract infections, particularly in developing countries [1]. UTI is a common and important clinical issue that warrants attention among children and parents [2]. Infants and young children may come with nonspecific signs and symptoms, including fever and fussiness and occasionally parental reporting of irritability and foul-smelling urine [3]. Untreated UTIs in children can have long-term consequences, such as renal scarring, hypertension and end-stage renal disease [4].

It is one of the most frequently treated microbial illnesses visited in ambulatory care facilities annually in developed countries [5]. UTI in children accounts for nearly 1% of physician office visits and 5–14% of emergency visits in children annually. A large meta-analysis concluded a varied prevalence rate of UTI by age, gender, race and circumcision status. Uncircumcised male infants less than 3 months and female infants younger than 12 months had the highest prevalence rate of UTI [6,7].

Bacterial infections are the most common cause of UTI, accounting for more than 90% of the cases. Gram-negative organisms are most commonly isolated from urine samples of children, with *Escherichia coli* (*E. coli*) accounting for almost half of the isolates [8,9].

Irrational use of antibiotics nowadays has resulted in an emerging increase in antibiotic resistance among UTI pathogens in children [9]. The resistance was found to be highest for oral drugs compared with intravenous antibiotics.

Pediatric UTI has been associated with short-term and long-term morbidity, highlighting the importance of prompt diagnosis and early treatment. Antibiotic resistance in Gram-negative Enterobacteriaceae has increased dramatically worldwide during the last two decades, highlighted by the development of extended-spectrum beta-lactamase (ESBL) producing organisms. The resistance patterns of commonly reported uropathogens are worrying in many African countries, including Somalia, where uncontrolled antibiotic prescribing is common [6,9]. Studies concerning the epidemiology and drug susceptibility pattern of UTI in children in developing countries are scarce. Moreover, no studies in the literature concerning pediatric UTIs in Somalia exist. This study aimed to evaluate the antimicrobial resistance pattern and uropathogens distribution in children at a referral hospital in Mogadishu, Somalia.

## Methods

A four-year retrospective study was conducted at Mogadishu Somali Turkish Recep Tayyip Erdogan Training and Research Hospital from 2019 to 2022. The study included 840 participants in all pediatric age groups whose urine had been cultured throughout the study period. Patients with catheter associated UTI, neurogenic bladder, those with diabetes and chronic renal failure were excluded from the study.

Investigated parameters were age (between 1 month and 18 years), gender, admission source (outpatient clinic, inpatient and intensive care unit), urine culture results and antimicrobial susceptibility and resistant pattern. Antimicrobial resistance to at least one antibiotic drug in three or more antimicrobial classes (extended-spectrum penicillins, cephalosporins, fluoroquinolones, carbapenems and aminoglycosides) was considered a multidrug-resistant uropathogen.

The legal guardians were typically provided with suitable guidance on the collection of midstream clean catch urine, which was instantly transferred to the microbiology unit for processing.

An antimicrobial susceptibility test was carried out using the standard Kirby-Bauer disk diffusion method based on the recommended Clinical and Laboratory Standards Institute (CLSI) system (10). Positive culture results were considered for plates showing 100,000 colony-forming units (CFU)/ml. The microorganisms were identified using eosin methylene blue agar and blood agar. A double-disk diffusion test DDST for the assay of ESBL production (11). The production of extended-spectrum beta-lactamases (ESBL) was recognized from urine samples that show growth to differentiate the patterns of growth between antibiotics with a lactamase inhibitor and those without using cephalosporins (ceftriaxone (30 μg), +cefotaxime (30 μg), +ceftazidime (30 μg)) and amoxicillin–clavulanic acid (20/10 μg) as the identifying disc. Disk diffusion zone of growth inhibition and mic values (susceptible (S) and resistant (R) categories) for antimicrobial susceptibility testing (AST) according to the CLSI criteria [8].

Mueller-Hinton agar was used to assess antimicrobial sensitivity and resistance. The antibiotic susceptibility of uropathogens was studied against piperacillin/tazobactam (100/10 μg), meropenem (10 μg), ertapenem (10 μg), colistin (10 μg), amikacin (30 μg), tigecycline (15 μg), cefepime (30 μg), cefazolin (30 μg), ceftazidime (30 μg), piperacillin (100 μg), linezolid (30 μg), clindamycin (2 μg), penicillin (G 1 U), trimethoprim/sulfamethoxazole (1.25/23.75 μg), vancomycin (30 μg), daptomycin (30 μg), tetracycline (30 μg), erythromycin (15 μg), cefoxitin (30 μg), ciprofloxacin (5 μg), nitrofurantoin (300 μg) and teicoplanin (30 μg) (8).

This study was approved by the ethical committee board of Mogadishu Somalia Turkish Training and Research Hospital (REF. MSTH-10617). Informed consent was not required since it was a retrospective study.

Data was entered and analyzed using the IBM SPSS Statistics 26 version and the results were expressed as percentages. Descriptive statistics were computed for most study variables and frequency distribution tables were used to describe the findings. The chi-square test and cross-tabulations were used to detect the significant association between the variables. A p-value of <0.05 was considered statistically significant.

## Results

A total of 840 urine cultures were performed over 4 years period. Of these, 148 were shown bacterial growth. The prevalence of culture-proven pediatric urinary tract infection (PUTI) was 17.6%. Females slightly predominated the study populations, accounting for 55.4%(n = 469), while boys were 44.6%(n = 371) of the cases. Regarding the age group of the patients, 12–18 years was the predominant age group (29.8%, n = 250), followed by 1–5 years (26.0%, n = 218). Infants (<1 y) accounted for (18.5%, n = 155). There was a slight difference regarding bacterial growth in the cultures and gender (17.3% for males and 18.0% for females) (Table 1). Of 148 cases that showed bacterial growth, 73, 54 and 21 were patients from the outpatient department, ward and pediatric intensive care unit, respectively.

**Table 1.** Culture results according to age, gender and type of patients.

Variable	Total, %	Culture-negative	Culture-positive
Age <1 y 1–5 y 6–11 y 12–18 y	155 (18.5%) 218 (26%) 217 (25.8%) 250 (29.8%)	127 175 184 206	28 43 33 44
Gender Female Male	469 (55.8%) 371 (44.2%)	388 304	82 66
Type Outpatient İnpatient unit PICU	398 (47.3%) 298 (35. 5%) 144 (17.1%)	325 244 123	73 54 21

*E. coli* was the most common pathogen isolated from the cultures, accounting for 48%, followed by *Klebsiella pneumoniae* (16.2%), *Pseudomonas aeruginosa* and *Staphylococcus aureus*, about 7.4% each (Table 2). Approximately 74.3%(n = 110) of the pathogens belonged to Gram-negative bacteria, while 16 cases (10.8%) were Gram-positive. Sixteen cultures (10.8%) showed ESBL-producing pathogens and *K. pneumoniae* was the predominant pathogen in 10/24 cases, followed by *E. coli* in 5/71 patients. About 27% (n = 40) of the pathogens had a multidrug-resistant (MDR) pattern. *K. pneumoniae* was associated with the highest MDR pattern at 45.8%, followed by *E. coli* at 32.3% and *P. aeruginosa* at 27.3%. Twenty-two cases had the *Candida* species.

**Table 2.** Distribution of uropathogens that showed bacterial growth according to age and gender.

Pathogen	Total, %	Age				Gender	
		<1 y	1–5 y	6–11 y	12–18 y	Male	Female
*E. coli*	71 (48%)	12	23	16	20	38	33
*K. pneumoniae*	24 (16.2%)	8	3	7	6	12	12
*P. aeruginosa*	11 (7. 4%)	1	6	2	2	8	3
*S. aureus*	11 (7. 4%)	1	4	1	5	6	5
*Enterococcus*	4 (2.7%)	1		1	2	1	3
*Proteus mirabilis*	2 (1.3%)		1		1	2	
*Candida*	22 (14.8%)	5	6	3	8	11	11
Others	3 (2%)			2	1	2	1

Most of the cephalosporins class (83%) had shown a higher resistance rate against the pathogens (Table 3). Nitrofurantoin (24.6%), Fosfomycin (15.2%) and Trimethoprim-sulfamethoxazole (SMX-TMP) (79.7%) encountered an increasing resistance rate against community and nosocomial-acquired UTIs. Carbapenems revealed an antimicrobial resistance rate ranging from 6.8 to 13.6%. The highest sensitivity rate (100%) was observed in tigecycline, teocopalmin, daptomycin, colistin, vancomycin and linezolid. *K. pneumoniaee* (81.5%) had higher resistance against cephalosporins than *E. coli* (57.9%). Nitrofurantoin, Fosfomuycin and SMX-TMP showed an antimicrobial resistance rate of 15.6, 15.6 and 77.8% versus 41, 14.3 and 82.3% against community and nosocomial-acquired UTIs.

**Table 3.** The antimicrobial resistance pattern against individual pathogens.

Medication	Total/resistant	*E. coli*	*K. pneumoniae*	*P. aeruginosa*	*S. aureus*
Cephazolin	45 (77.8%)	75.8%	84.6%	50%	
Cefotaxime	29 (89.6%)	84.2%	100%		
Cefoxitin	36 (55.6%)	45%	87.5%	100%	20%
Cefuroxime	77 (77.9%)	76.3%	88.8%		
Ceftriaxone	33 (75.8%)	78.3%	87.5%		
Ceftazidime	24 (54.2%)	60%	100%	37.5%	
Cefipime	11 (36.3%)	0%	50%	50%	
Cefixime	48 (70.8%)	71.5%	80%		
Ampicillin	75 (95%)	93.6%			
Cefoparozin-sulbactam	28 (35.7%)	30%	57.1%		
Piperacillin	13 (61.5%)	20%		87.5%	
Piperacillin-Tazobectum	54 (16.6%)	7.1%	45.5%	18.8%	
Amoxicillin-clavulanic acid	63 (41.3%)	41. 4%	35.7%		
Gentamycin	56 (30.3%)	30%	27.3%	50%	50%
Amikacin	99 (4%)	3.3%	5%	11.1%	
Smx-tmp	79 (79.7%)	82%	81.3%	100%	40%
Ciprofloxacin	74 (54.1%)	61.9%	58.3%	33.3%	33.3%
Levofloxacin	40 (40%)	38.3%	33.3%	50%	75%
Nitrofurantoin	65 (24.6%)	22.2%	38.5%	50%	
Fosfomuycin	46 (15.2%)	20.6%	8.3%	100%	
Imipenem	58 (6.8%)	0%	13.3%	40%	
Meropenem	66 (13.6%)	5.1%	30%	30%	
Ertapenem	53 (13.2%)	10.8%	0%		100%
Linezolid	11 (0%)	0%	0%	0%	0%
Vancomycin	13 (0%)	0%	0%	0%	0%
Tigecycline	19 (0%)	0%	0%	0%	0%
Teocopalmin	8 (0%)	0%	0%	0%	0%
Daptomycin	9 (0%)	0%	0%	0%	0%
Clindamycin	10 (20%)				22.2%
Ampicillin-sulbactam	4 (50%)				
Colistin	20 (0%)	0%	0%	0%	0%

## Discussion

Urinary tract infection is among the most prevalent infectious diseases, with a considerable financial burden on the community, especially in low-income countries. Thus, prompt diagnosis of UTI in children is crucial [7]. In our study, the prevalence of culture-proven PUTIs was 17.6%. It is in line with a study done in Northwest Ethiopia, which showed an overall prevalence of 16.7%, a study from Tanzania, which revealed a prevalence of 16.8% among children who presented prolonged duration of fever (7 days or longer) and another study from Nepal, which documented 15.88% [12,13]. Lower rates of between 7 and 11% have been reported in a survey of the prevalence of UTI in two Egyptian teaching hospital, a hospital-based study from South Africa and another study in Kenya on the contribution of urinary tract infection to the burden of febrile illnesses in young children [^14-16^]. Higher prevalence rates of pediatric UTIs were noted in two studies from Ethiopia at about 26.5 and 27.5% and another study in India also showed a 48% prevalence [12,17]. The variation in reported rates of UTIs in the studies might be due to differences in study design and sample size, the difference in the age of subjects and the ritual of personal hygiene.

UTI prevalence varies by age, gender and geographical area. Up to 7 years of age, 8% of girls and 2% of boys will have at least one episode of urinary tract infection [7]. UTIs are more common in females, likely due to anatomical variations, hormonal impacts and behavioral factors. The current study showed a slight difference regarding bacterial growth in the cultures and gender (17.3% for males and 18.0% for females).

The rate of Gram-negative bacteria in our study was 74%, corresponding to most studies conducted in the world; the commonest uropathogens were Gram-negative bacteria, with *E. coli* being the leading pathogen [18]. The frequency and pattern of isolation of pathogens vary with different geographical areas. Due to their increased antimicrobial resistance, *E. coli* and *K. pneumoniae* have emerged as serious pathogens worldwide, resulting in substantial morbidity and healthcare-related costs. *E. coli* was the most common pathogen isolated from the cultures, followed by *K. pneumoniae* in our study. This is in line with another study done in South-East of Gabon which was found *E. coli* as leading cause of UTI, followed by *Klebsiella pneumonia* [19]. *E. coli* is the most common uropathogen responsible for UTIs irrespective of age, gender and community-acquired and hospital-acquired UTIs, account for 50–90% of cases [20]. It originates from the fecal flora, spreads across the perineum and invades the bladder through the urethral meatus in ascending fashion. In recent years, Gram-positive organisms have become an important cause of UTIs, although Gram-negative bacteria are responsible for most cases [6].

We observed an alarmingly high rate of 27% prevalence of MDR uropathogens (antimicrobial resistance to at least one antibiotic drug in three or more antimicrobial classes (extended-spectrum penicillins, cephalosporins, fluoroquinolones, carbapenems and aminoglycosides).

This coheres with rates reported by Shrestha and colleagues [20]. Belete and associates reported higher MDR rates of more than 90%; 66% MDR prevalence was observed by Fenta *et al.* [12,18]. Widespread misuse of antibiotics, inappropriate prescription of drugs, easy availability of antimicrobials, sub-standard/poor drug quality, incompletion of the standard dosage and lack of knowledge about drug resistance are principal leading factors for emerging increased antimicrobial resistance.

Widespread antimicrobial resistance is rising against the Enterobacteriaceae family; notably, *E. coli* and *Klebsiella*, a public health threat, consistently exhibited higher resistance levels in children worldwide [21,22]. Due to the rapid emergence of antimicrobial resistance (AMR), infection and mortality rates associated with AMR are constantly increasing. The estimated incidence of antimicrobial resistance infections in UK increased from 61,946 patients in 2018 to 65,162 individuals in 2019 [23,24]. On the contrary, the European Centre for Disease Prevention and Control (ECDC) has documented that the annual incidence of antimicrobial resistance (AMR) in the European Union (EU) exceeds 670,000 cases [25]. Based on an analysis of data from a previous study, bacterial AMR was responsible for 4.95 million fatalities globally in 2019, of which 1.27 million were directly attributable [26]. A review recognized the projection that by 2050, the annual mortality toll directly attributable to antimicrobial resistance (AMR) would increase to 10 million. Asia is estimated to have the highest number of fatalities, followed by Africa. This is primarily due to the continent's large population and the absence of regulations concerning AMR prevention [27]. According to the Global Burden of Diseases (GBD) region associated explicitly with antimicrobial resistance (AMR), sub-Saharan Africa exhibits the highest all-age mortality rate [26]. In contrast, Australasia recorded the lowest mortality rate related to AMR in 2019. Antimicrobial resistance against these pathogens decreases antibiotic choices, increasing healthcare expenditures and mortality [28]. In the community and nosocomial UTIs, the causative pathogens and the antimicrobial resistance patterns have changed over the past years. Due to the geographical and historical heterogeneity of antimicrobial resistance patterns, it is vital to comprehend the sensitivity pattern of common uropathogens based on local epidemiological studies to choose an appropriate antibiotic for empirical treatment. In addition, studies suggest that policies should be reevaluated every 5 years based on local resistance rates [22]. Godman and colleagues studied Strategies to Improve Antimicrobial Utilization with a Special Focus on Developing Countries [29]. They reported that an increasing number of factors contribute to the rise in AMR, including the rising utilization of antimicrobials, particularly in LMICs [30,31]. Antimicrobial overuse has been linked to an increase in AMR [32,33]. Furthermore, AMR rates are exacerbated by the availability of substandard, counterfeit or falsified antibiotics, high rates of self-medication, particularly in least-developed countries (LMICs), frequently for self-limiting diseases, the extensive use of antimicrobials to prevent and treat diseases in animals even though their manure is frequently used in aquaculture and food production, water pollution, improper sanitation and hand hygiene and international travel.

Urinary tract infections in children are linked to a high-risk rate of morbidity and long-term consequences such as renal abnormalities, septicemia, renal scarring, hypertension and chronic renal failure [34,35]. To avoid these consequences, early detection and antibiotic treatment are necessary.

Identifying uropathogen characteristics and patterns of antimicrobial sensitivity and resistance is paramount in the effective management and determination of empiric treatment for patients presenting with urinary tract infections (UTIs). Ongoing evaluation is essential to monitor the evolving patterns of antimicrobial sensitivity and resistance to uropathogens [28].

Despite the goal of this study to provide a comprehensive review of the epidemiology and characteristics of pediatric UTIs and examine resistance patterns in isolated organisms, this study has certain limitations, including a lack of data regarding previous antimicrobial use and hospitalization. Predisposing factors were not emphasized and further studies are needed to explore its association with increasing MDR rate. Although this study is the first study reported from Somalia, it is a single-center-based study with a small sample size.

## Conclusion

This study revealed the burden of pediatric urinary tract infection, major bacterial uropathogens and their antimicrobial susceptability pattern. *E. coli* and *K. pneumoniae* were the most common pathogens isolated from the cultures and consistently exhibited higher resistance levels. High rate of multidrug-resistant organisms were identified in this study, it is therefore suggested that there has to be a high index of suspicion and appropriate antimicrobial selection to reduce the risk of MDR and improve the effectiveness of antibiotics. This widespread antimicrobial resistance decreases antibiotic choices and increasing healthcare expenditures. Continuous surveillance, monitoring and reporting of local antimicrobial resistance patterns are paramount for identifying appropriate empirical antibiotic therapy before the availability of culture results.
